# A nodulo-cystic eumycetoma caused by Pyrenochaeta romeroi in a renal transplant recipient: A case report

**DOI:** 10.1186/1752-1947-5-460

**Published:** 2011-09-14

**Authors:** Umasankar Mathuram Thiyagarajan, Atul Bagul, Michael L Nicholson

**Affiliations:** 1 Department of Infection, Immunity & Inflammation. Transplant Group, University of Leicester, Leicester General Hospital, Gwendolen Road, Leicester, LE5 4PW, UK

## Abstract

**Introduction:**

*Pyrenochaeta romeroi *(*P. romeroi*) is a saprophytic fungus found in soil and plants. The fungal spores can be introduced into deeper tissues by trauma. It causes eumycetoma, which affects skin and subcutaneous tissues.

**Case presentation:**

A 57-year-old South Asian man presented with a painless, nodular lesion (1 cm × 0.5 cm) on the left knee. He had had a renal transplant eight months earlier for end-stage renal failure. The patient was on tacrolimus, mycophenolate mofetil and prednisolone for immunosuppression. The lesion had progressed dramatically (to 5 cm × 5 cm) despite antibiotic treatment. The size and location of the lesion was severely affecting his quality of life, so an excision biopsy was performed. Nuclear ribosomal repeat-region sequencing confirmed the causative organism as *P. romeroi*. An *in vitro *antifungal susceptibility test demonstrated that *P. romeroi *was sensitive to voriconazole. Following a successful surgical removal, voriconazole was continued orally for two months.

**Conclusion:**

To the best of our knowledge, we are reporting the first case of Eumycetoma caused by *P. romeroi *in a renal transplant recipient. Physicians should be aware of this rare fungal disease in transplant recipients. We recommend a combination of medical and surgical management in these immunosuppressed patients.

## Introduction

Eumycetoma is a chronic, specific, granulomatous, fungal disease involving cutaneous and subcutaneous tissue [1]. Only four cases of *P. romeroi *infection have been reported in the literature [2-5]. To the best of our knowledge, this is the first case in a renal transplant recipient. The majority of the reported cases of this disease have occurred between the latitudes of 15° south and 30° north [6]. It is extremely rare in temperate marine climates and sub-arctic zones such as Europe.

## Case presentation

A 57-year-old South Asian man presented with a painless, nodular lesion (1 cm × 0.5 cm) on his left knee. He had had a renal transplant eight months earlier for autosomal dominant polycystic kidney-related renal failure. He received two doses (20 mg) of interleukin receptor-2 antibodies (Simulect^®^, Novartis Pharmaceuticals, Surrey, UK) at induction and on the fourth postoperative day. This was followed by tacrolimus, mycophenolate mofetil and prednisolone for immunosuppression. During the follow-up period, his tacrolimus level was kept within the therapeutic range (5 to 8 ng/ml). He was cytomegalovirus (CMV) negative and had received a kidney from a CMV-negative deceased donor. He had immigrated to the United Kingdom from Bangladesh twenty-five years ago and had last visited there eight years ago. There was no past history of trauma to the knee.

**Figure 1:**
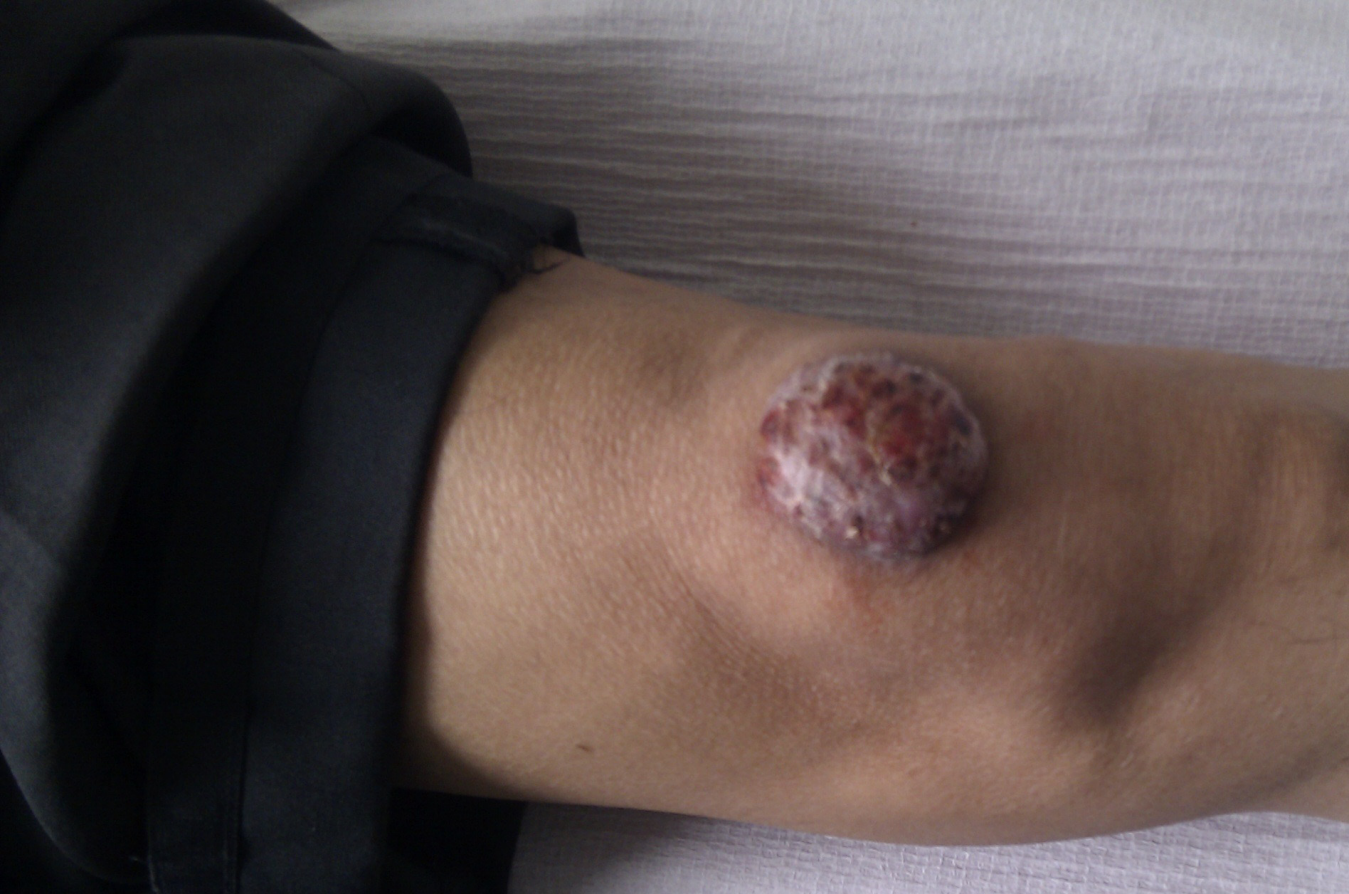
**Cystic lesion on the left knee**.

He was started on flucloxacillin and the course was extended for a period of 14 days. In spite of antibiotic therapy, the lesion progressed significantly and reached 5 cm × 5 cm (Figure 1) and became cystic in nature. The lesion was confined to the skin and subcutaneous tissue with no deep extension to bone or lymph node involvement. The surface of the lesion had multiple small sinuses showing a dark-brown discharge, which was sent for microscopy.

During this period, he did not show any systemic symptoms and inflammatory markers (C-reactive protein) and his white cell count were both normal. Because of the failure of antibiotic therapy and the fast growing nature of the lesion an incisional biopsy was performed.

Histological examination of the specimen showed marked epidermal hyperplasia and abundant fungal spores with hyphae. The microscopy of the discharge also confirmed the fungal spores and hyphae (fungal grains).

**Figure 2:**
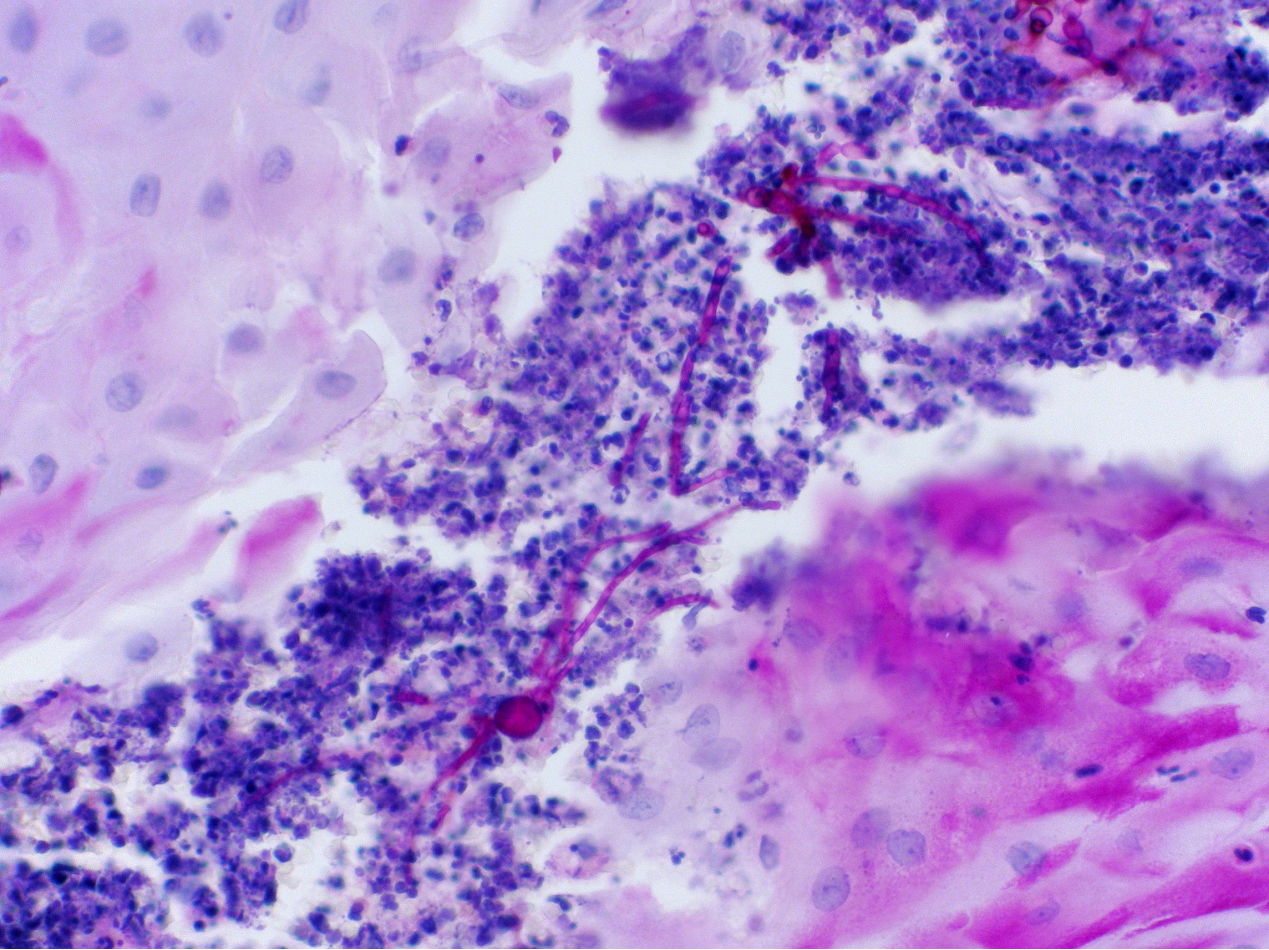
**PAS positive branching and septate hyphae on histology**.

The patient was started provisionally on voriconazole while awaiting confirmation of the causative organism. The large size of the lesion on his knee significantly reduced his quality of life so an excision biopsy was done. The specimen showed marked epidermal hyperplasia with microabscess formation. Within the microabscess, there were PAS (Periodic acid-Schiff) positive branching and septate hyphae (Figure 2). No bacteria were found. The excision margins were clear, and no evidence of the neoplastic process was found.

Nuclear ribosomal repeat-region sequencing confirmed that the causative organism was *P. romeroi *(Mycology Reference Laboratory, Bristol, UK). An *in vitro *antifungal susceptibility test demonstrated that *P. romeroi *was sensitive to voriconazole. Following a successful surgical removal, voriconazole was continued orally for two months under the care of the infectious-disease team. No recurrence was seen during the following six months.

## Discussion

Eumycetoma can cause skin and deep-tissue involvement and may result in the need for an amputation [6]. *P. romeroi *is a saprophytic fungus found in soil and plants that can be introduced into deeper tissues by trauma [7]. A typical case of eumycetoma usually presents in exposed areas of the body but it is not uncommon in unexposed areas. The exact reason for infection in unexposed areas is unknown but it may be related to the bacterial count and moist skin [8]. *P. romeroi *and *P. mackinnonni *have been isolated from clinical material obtained from patients with mycetoma [9,10]. The lesions were found to develop slowly following trauma and they were usually localized to cutaneous and subcutaneous tissues [11].

The identification of this organism was difficult because of the inability of some strains to readily produce characteristic diagnostic structures in cultures and also because of a lack of expertise in diagnostic microbiology laboratories. Young *et al*. [12] reported a similar case in a transplant recipient but the histopathological appearance of this lesion was closely similar to those caused by *Exophiala jeanselmei *or *Phialophora richardsiae*. Microscopy showed dematiaceous hyphae with yeast-like cells and the diagnosis was a phoma-like species resembling *P. romeroi*.

Girard *et al*. [3] reported a *P. romeroi *infection in a leprosy patient but to the best of our knowledge our patient is the first confirmed case in an immunocompromised renal transplant recipient. Our case was reported during the eight months after transplant. The immunosuppression was achieved with tacrolimus (therapeutic level maintained at 5 to 8 ng/ml), mycophenolate mofetil and prednisolone at the time of diagnosis. The immunosuppressive therapy was within the unit protocol, and he never had any viral infection during this period. Although the infection can be coincidental, previous cases have been reported mostly in immunocompromised patients. In our patient, the role of the immunosuppressive burden was likely to be a precipitating factor. Hence, these patients need early and thorough examination to avoid further morbidity and mortality.

Because these organisms are seen in tropical/sub-tropical countries, we cannot explain the possible source and mode of transmission of *P. romeroi *to our patient. The spores may have been introduced in the past and remained dormant until he was started on immunosuppression. This theory of the dormant nature of spores is also supported by another case report where the infection developed three years after immunosuppression was started for leukemia [5]. Hence, it is important to note any history of immigration and foreign travel.

The literature shows that from a treatment point of view, surgical treatment is usually successful because no standardized therapy is available [13]. The literature also suggests that antifungal susceptibility of *Pyrenochaeta *species is scanty due to low availability and the low number of clinical isolates [2,4].

*P. romeroi *has been shown to be resistant to broad-spectrum antifungals such as amphotercin B, fluconazole, itraconazole and caspofungin [2,4]. Khan *et al*. [5] reported that *P. romeroi *is sensitive to voriconazole and posaconazole and a similar susceptibility was found in our case. The treatment should be tailored according to the location and size of the lesion. In areas over joints and bones, it is worth combining surgical and antifungal treatments. This can be curative and can prevent early deep-tissue involvement.

Khan *et al*. [5] have reported a single case of subcutaneous infection in a patient with acute lymphoid leukemia (ALL) who was successfully treated with surgical excision and required no medical management. Other reports [2,4,14] suggest using surgical debridement followed by prolonged use of triazole, which we also feel is reasonable because it reduces the risk of disseminated infection in immunosuppression.

## Conclusions

It is imperative to consider a fungal etiology when an infection is not responding to antibiotics in post-transplant patients, even if the patient is living in a geographical area where this fungus is uncommon. Early diagnosis can be made by incision biopsy, but surgical excision should be considered if the infection is over the bone or joints. We recommend a combination of surgical and extended antifungal treatment in post-transplant immunosuppressed patients to prevent invasive disease.

## Consent

Written informed consent was obtained from the patient for publication of this case report and any accompanying images. A copy of the written consent is available for review by the Editor-in-Chief of this journal.

## Competing interests

The authors declare that they have no competing interests.

## Authors' contributions

UMT and AB were involved in drafting the manuscript. MLN revised the manuscript. All authors have read and approved the final manuscript. All three were involved as part of team management.
